# The psychological challenges for oncological patients in times of COVID-19 pandemic: telemedicine, a solution?

**DOI:** 10.2217/fon-2020-0552

**Published:** 2020-07-13

**Authors:** Estelle Caroline Schade, Ronaldo Elkaddoum, Hampig Raphael Kourie

**Affiliations:** ^1^Faculty of Psychology, University of Vienna, Vienna, Austria; ^2^Department of Hematology-Oncology, Faculty of Medicine, Saint Joseph University of Beirut, Beiurt, Lebanon

**Keywords:** cancer-related distress, COVID-19, patient, psycho-oncology, psychology, telemedicine

City lockdowns around the globe were imposed with the outbreak of COVID-19 pandemic, a respiratory infection caused by SARS-CoV-2. While symptoms of this disease are usually mild to moderate, cancer patients are keener to develop complications and are among the groups with the highest mortality rates [1]. When the danger of contracting COVID-19 outweighs the benefits of a physical visit to the care facility, telemedicine in oncology (teleoncology) is widely encouraged [2]. From another side, a third of oncological patients may be psychologically affected during their treatment thus needing professional support [3]. While guidelines, advantages and setbacks of teleoncology in times of the pandemic have been discussed, the psychological aspect of it has not yet been put under the spotlight. In our article, we review the challenges of cancer patients’ mental health in times where their concerns about risks, treatments and privacy grow bigger. We also discuss the psychological benefits of teleoncology and the importance of empathy in creating a patient-centered approach. We conclude by suggesting improvements applicable to teleoncology practice during the COVID-19 pandemic.

Cancer patients are subject to psychological effects linked to their condition. Indeed, cancer-related distress, intense unpleasant emotions that interfere with functioning, has been listed as a common negative consequence of a diagnosis and is closely linked to uncertainty [4]. Detecting and preventing distress is especially crucial as 10–20% feel chronic distress, sometimes up to 6 years after the end of the treatment [3]. The challenges for cancer patients in the specific context of COVID-19, add to their preexisting psychological burden, especially since cancer has a big share (20%) in COVID-19 mortality rate [1]. A factor of uncertainty also lies in the controversies on whether anti-cancer treatments, such as immunotherapy, interfere with COVID-19 [5]. Not knowing if the treatment they take may disadvantage them in case they contracted COVID-19 will put them in much more distress than the average population. Furthermore, cancer patients are considered a higher risk-group and have specific recommendations against COVID-19. For example, social distancing or keeping 1.5–2 m of distance from another person in order to avoid their respiratory droplets, is preconized in many countries and particularly for cancer patients. While this helps reduce their contamination, it negatively impacts their emotions and may add to their distress. For example, in recommendations for palliative care [6], mild pain, lower than 7/10, is not a high priority to look at. Therefore, patients may be physically suffering while isolated. Second, the urgent need for therapy at the hospital is an added factor of confusion since it contradicts the preconized physical distancing [7]. Also, with the suspension of flights during the pandemic, cancer patients who usually seek care overseas with more advanced technologies, see their treatment plans compromised. This increases the psychological burden on patients who are aware that the later they are getting treated, the worse their prognosis could get. Moreover, in normal times, cancer patients are among those who are admitted the most to the ICU, either for sepsis or respiratory difficulties [8]. However, with COVID-19, ICUs are being overwhelmed with the raise of patients infected with the virus [9]. The scarcity of ICU beds is forcing physicians to choose between allocating the beds to COVID-19 patients and other patients, oncological ones included. The news of triage where old cancer patients are less likely to be prioritized in ICUs might have a heavy impact on patients’ morale and their will to fight cancer. Thus, the consequences of the COVID-19 pandemic, concerning physical contact and medical appointments, can increase the feeling of uncertainty and distress, making it particularly important to address those issues [7].

Parts of this uncertainty, such as information related uncertainty, can be remediated through good patient–physician communication, which might prevent the uncertainty from turning into distress [4]. While patient–physician communication is compromised due to isolation measures, teleoncology could offer a solution. Teleoncology, is the use of various technologies, such as video calls (e.g., Whatsapp, Zoom, etc.) or health applications (e.g., MyChart) that allow the oncologists to screen, diagnose, treat, follow-up and support patients at a distance. At the beginning, it intended to reduce disparities in the access to oncological care [10]. Over the years, many recommendations [11] and reports have arisen. From the increase of colorectal cancer screening for risk groups [12], to psychological behavioral therapy for breast cancer survivors using video conferencing [13], passing by the successful remote administration of chemotherapy [14] and the inclusion of non-small-cell lung cancer patients in multimodality therapies’ clinical trials [15]. While teleoncology seems to hold many promises, until the beginning of the year it was rarely used out of the context of experimentations. With the restrictions following the course of the COVID-19 pandemic, teleoncology, that is considered at least equivalent to physical clinical care [10], is recommended while not neglecting ethical considerations.

The first concern with teleoncology lays in its’ accessibility. Cancer patients who are usually older than the general population may lack the knowledge to connect to a video consultation while patients from low-income families may lack the financial means to do so. A second concern is that online sharing of information can jeopardize patients’ privacy, especially in times of COVID-19, where more health data is transferred online, thus raising the interest of hackers in physicians’ accounts. Consequently, patients fear that information they give may reach unwanted parties.

On a brighter note, with teleoncology, patients can receive appropriate care at a distance. In the past they had to travel long distances to get to specialized centers, a high price to pay for personalized therapies. This did not only affect diagnosis and treatment but the quality of life too [16]. Teleoncology reduces stressful travel, thus eventually improving their mental health. With COVID-19 and the switch to telehealth, the time spared from commuting between care centers, can make the oncologists keener to actively listen and give detailed explanations to patients and involve them in the decisional process. This creates a patient-centered communication, which is known to positively impact survival [17]. It also provides a more comprehensive approach, including emotional and psychological support.

## Suggestions

### Advice for the patients

Support groups consist of cancer patients or survivors who share their experiences and information. They usually give nondiscriminative moral support and serve as a shield against isolation and loneliness [4]. They remain crucial, even for patients who do not need psychotherapy. The COVID-19 pandemic limits physical support group meetings. Oncologists should encourage their patients to organize and attend virtual group meetings, using video conferencing (Zoom, Skype, etc.) as soon as possible since technical barriers are minimal.

### Advice for the oncologists

Oncologists need to keep in mind that their patients might still be lacking the support that they previously benefited from. Consequently, they need to be even more sensitive to the concerns of their patients and the challenges those might face. Those challenges can include distress and aspects that can lead to it, such as residual physical symptoms, the quality of the support system around the patient and previous coping mechanisms. We suggest two screening tools to evaluate distress and facilitate early intervention. One is the distress thermometer that was developed by the National Comprehensive Cancer Network [18]. The other one is the emotion thermometer developed by Hinz and colleagues [19]. Both are free, accessible online, available in several languages and only require a few minutes. Oncologists can thus easily administer them to the patients during the video conference.

Finally, empathy plays an important role in the interaction between healthcare professionals and patients. An empathic interaction requires a patient to send socio-emotional cues to share an affective state, the physician to feel and convey empathy and the patient to perceive this empathy. However, video consultations can cause filtering effects that prevent the perception of those socioemotional cues, meaning that empathy might either not be shown or simply not perceived [19]. To remediate the loss of signals needed for an empathic interaction and thus improve the quality of telecommunication, different strategies are immediately implementable by the oncologists in interaction with their patients.

First, there are technicalities, such as finding a quiet place with a stable connection, setting the webcam as close as possible to the patient's image on the screen for a better eye contact, while also showing the whole upper body of the oncologist.

Second, to facilitate the perception of socioemotional cues of the patients, attention should be payed to vocal tone variations and they should be encouraged to exaggerate facial expressions. This last point is also important for the physician to show affective resonance.

Lastly, empathy can be conveyed by matching the language style of the patient, showing body gestures that are congruent with the affective state of the patient, maintaining eye contact and an active posture, making positive statements and asking open questions [20].

## Conclusion

In conclusion, teleoncology is challenging from a mental health perspective. Nevertheless, few adjustments in its presentation and modality can make it more acceptable to the patients and teleoncology may even become a new norm in the coming years. While medical and psychological issues are being tackled at a fast rate, the main question is whether governments will be able to provide access to telehealth without racial, sexual, religious, regional or economical discrimination.
